# Disparities in the Healthfulness of School Food Environments and the Nutritional Quality of School Lunches

**DOI:** 10.3390/nu12082375

**Published:** 2020-08-08

**Authors:** Sarah Bardin, Liana Washburn, Elizabeth Gearan

**Affiliations:** 1 Mathematica, 955 Massachusetts Avenue, Suite 801, Cambridge, MA 02139, USA; lgearan@mathematica-mpr.com; 2 Mathematica, 1100 First Street, NE, 12th Floor, Washington, DC 20002, USA; lwashburn@mathematica-mpr.com

**Keywords:** school food environment, nutritional quality, Healthy Eating Index-2010, School Nutrition and Meal Cost Study, National School Lunch Program, disparities, race, free and reduced-price lunch

## Abstract

The Healthy, Hunger-Free Kids Act (HHFKA), a public law in the United States passed in 2010, sought to improve the healthfulness of the school food environment by requiring updated nutrition standards for school meals and competitive foods. Studies conducted since the passage of the HHFKA indicate improvements in the food environment overall, but few studies have examined whether these improvements varied by the socioeconomic and racial/ethnic composition of students in schools. To better understand the extent of disparities in the school food environment after HHFKA, this paper examined differences in the healthfulness of school food environments and the nutritional quality of school lunches by the school poverty level and racial/ethnic composition of students using data from the School Nutrition and Meal Cost Study. Results from chi-square analyses showed lower proportions of high poverty, majority black, and majority Hispanic schools had access to competitive foods, while higher proportions of these schools had a school wellness policy in addition to a district wellness policy. The overall nutritional quality of school lunches, as measured by total Healthy Eating Index (HEI)-2010 scores, did not vary significantly across school types, although some HEI component scores did. From these findings, we concluded that there were disparities in the school food environment based on the socioeconomic and racial/ethnic composition of students in schools, but no significant disparities in the overall nutritional quality of school lunches were found.

## 1. Introduction

Children in the United States spend a significant portion of their waking hours at school and consume more than one-third of their total daily calorie intake while there [1]. Research has shown that schools play an integral role in shaping the dietary behaviors of children [2,3,4]. As a result, policymakers and practitioners have viewed the school food environment as a critical setting for influencing children’s eating behaviors and reducing childhood obesity [5,6,7]. In communities with less access to healthy food, schools may provide a particularly important opportunity to positively impact student’s health, especially as research has shown that living in low-income and minority communities is linked to poorer diet quality for children [8,9,10], which in turn can lead to adverse health outcomes [11,12,13].

The school food environment includes both the availability of food items and the school food policies that regulate their accessibility and aim to promote healthy food choices [2,14]. Most schools provide students with access to meals through the National School Lunch Program (NSLP) and the School Breakfast Program (SBP), both administered by the United States Department of Agriculture (USDA). To receive reimbursement from the USDA for meals served to students, the meals must meet nutrition standards that specify the types and amounts of foods to be offered. In many schools, students also have access to competitive foods sold to students outside of reimbursable meals [15]. Schools can offer competitive foods in a variety of settings, such as through a la carte sales in cafeterias during mealtimes, vending machines, school stores, snack bars, food carts/kiosks, or fundraisers.

The Healthy, Hunger-Free Kids Act (HHFKA) required several changes to nutrition standards and school food policies to improve the healthfulness of school food environments [16]. Specifically, this legislation led to updated nutrition standards for reimbursable meals designed to improve the nutritional quality of the meals and better align them with the Dietary Guidelines for Americans (DGA). It also introduced new nutrition standards for competitive foods (Smart Snacks in School) [17] and strengthened and expanded the scope of school wellness policies [18]. Although research suggests these changes have had positive effects on the nutritional quality of school meals selected and consumed by students [19,20,21,22,23], studies have also shown that schools have experienced challenges in adopting the updated nutrition standards, particularly with finding products that comply with sodium and whole grain requirements [15,24].

Since the implementation of these new standards, research has shown that socioeconomic and racial/ethnic disparities in the healthfulness of school food environments have declined [25,26]. However, these studies focused on only one dimension of the school food environment—the availability of food items—and included only a small number of grade levels in their samples, leaving a limited understanding of the degree to which disparities exist across the nation’s schools. As a result, a more thorough examination of the healthfulness of school food environments is warranted. Using data from the School Nutrition and Meal Cost Study (SNMCS), this analysis explores potential disparities in greater depth by examining differences in the healthfulness of school food environments and the nutritional quality of NSLP lunches based on the poverty level and racial/ethnic composition of students in schools.

## 2. Materials and Methods

### 2.1. Study Design and Sample

The SNMCS was based on a nationally representative sample of 1282 public, non-charter schools that participated in the NSLP in school year 2014–2015. The study used a two-stage sampling approach to select public school food authorities (SFAs), and schools within SFAs, across the 48 contiguous states and Washington, DC. It sampled up to 8 schools within an SFA. Schools participated in several data collection activities that provided information on the school food environment and types and quantities of foods offered in NSLP lunches. This analysis is based on data from 1207 schools that completed the school-level instruments, which were the primary sources of information on the characteristics of school food environments and the composition of NSLP lunches.

The Office of Management and Budget and the New England Institutional Review Board reviewed and approved all data collection instruments. The study also complied with any institutional review process required by local school districts. Additional details regarding the study design, sampling, and data collection appear elsewhere [27,28].

### 2.2. Measures of the Healthfulness of School Food Environments

Several SNMCS instruments collected data related to the healthfulness of school food environments. These instruments included the School Nutrition Manager Survey and the SFA Director Survey, which respondents completed online, and collected information on the food environment, foodservice operating policies and practices, and nutrition promotion activities. In addition, competitive foods checklists, which onsite observers and school nutrition managers (SNMs) completed, provided data on the presence of competitive food sources in the school. After reviewing all available variables related to the school food environment, 17 binary (yes/no) variables were selected and then grouped into three domains that comprised the school food environment: the presence and sources of competitive foods, school food policies and programs, and nutrition promotion and outreach activities. The selection of these variables was informed by existing literature that has explored the healthfulness of school food environments [2].

The analysis used four variables to characterize the presence and sources of competitive foods: whether the school (1) offered any competitive foods; (2) sold foods other than milk on an a la carte basis; (3) sold foods in vending machines; and (4) sold foods in other venues, such as a school store, snack bar, food cart, kiosk, or fundraiser. Throughout this paper, the term *foods* refers to both foods and beverages.

Eight variables measured school food policies and programs; four of these variables were collected at the school level and four at the SFA level. The school-level variables included whether the school (1) operated a school garden, (2) participated in the Fresh Fruit and Vegetable Program (FFVP), (3) participated in their state’s Farm to School program, and (4) had a school wellness policy in addition to their SFA wellness policy. The SFA-level variables included whether schools in the SFA (1) had a pouring rights contract, which establishes a beverage company as a sole source vendor for beverages within the SFA or school; (2) offered foods through a national or regional brand-name or chain restaurant (branded foods); (3) had fully implemented the Smart Snacks in School standards, based on the Interim Final Rule which went into effect the summer before data collection began [17]; and (4) had competitive food standards that exceeded the Smart Snacks in School standards.

Finally, five variables characterized nutrition promotion and outreach activities conducted by the school: whether the school (1) shared information about its meal program with a nutrition advisory council, (2) involved students in planning menus, (3) conducted a nutrition education activity, (4) encouraged students to select fruit at meal times, and (5) sought students’ input into vegetable offerings.

### 2.3. Measure of the Nutritional Quality of NSLP Lunches

Data used to measure the nutritional quality of NSLP lunches came from Menu Surveys completed by SNMs or their designee. This instrument collected detailed information on all foods offered in NSLP lunches for one school week. This included food details needed for an accurate nutrient analysis, portion sizes, recipes, and the number of portions prepared for NSLP lunches. Foods reported on each school’s daily lunch menus were linked to two USDA databases: (1) the Food and Nutrient Database for Dietary Studies version 2011–2012 [29], which provided data on the energy and nutrient content of each food; and (2) the Food Patterns Equivalents Database version 2011–2012, which provided data on the USDA Food Pattern food group content of each food [30]. During data processing, the study used several procedures to ensure accurate estimates of the nutrient and food group content of the foods reported on schools’ menus. This included modifying database recipes for certain categories of foods to more accurately reflect the types and amounts of ingredients used by the schools and obtaining nutrient and food group profiles for specially formulated school food products from USDA’s Agricultural Research Service.

In each school, the nutrient and food group content of each daily lunch menu was estimated to reflect the amounts of each food that were actually prepared by the school and made available for students to select as part of an NSLP lunch. For each daily lunch menu, the nutrient and food group profiles for each food were weighted by the number of portions prepared and then summed across all foods reported on the menu. The daily sums were then divided by the number of NSLP lunches planned for the day to provide an estimate of the average nutrient and food group content of NSLP lunches prepared on the day. This approach gives greater weight to foods prepared in larger quantities. The daily averages were then averaged over the school week to estimate the nutrient and food group content of the average NSLP lunch prepared by the school. Additional details on procedures used to collect and process the Menu Survey data are available elsewhere [27,28,31,32].

The nutritional quality of the average NSLP lunch in each school was assessed using the Healthy Eating Index-2010 (HEI-2010). The HEI-2010 is a scoring metric that measures conformance with key recommendations of the 2010 DGA, which were in effect when the study data were collected [33]. The HEI-2010 has 12 components and a total score that provides an overall measure of nutritional quality. The HEI-2010 organizes components into two groups—9 adequacy components that focus on foods and dietary components that are encouraged for consumption, and 3 moderation components that focus on dietary components that should be limited or consumed in small amounts (Table 1).

Each component has a standard for achieving the maximum possible score and a standard for receiving the minimum score of zero (Table 1) [34]. The standards use a density approach (typically an amount per 1000 calories), which enables the HEI-2010 to assess the mix of foods and separate quantity from quality—meaning that NSLP lunches cannot achieve better scores by including larger quantities of food. Densities between the minimum and maximum standards are scored proportionately. Scores for each component are summed to construct the total HEI-2010 score, which has a maximum possible score of 100. Higher scores for each component and for the total score indicate higher nutritional quality and better conformance with DGA recommendations. This study estimated mean HEI-2010 scores for each school based on the average NSLP lunch prepared across the week.

### 2.4. Definition of Poverty and Race/Ethnicity Subgroups

To explore differences across school poverty subgroups, data on the percentage of students eligible for free or reduced-price lunch (FRPL) in each school were used to construct four poverty subgroups using the National Center on Education Statistics’ (NCES) definition [35]. The NCES definition was chosen as it is commonly used in education research to classify schools into meaningful subgroups for comparison [36,37,38]. This information came from the SFA Director Survey and was imputed for a small number of schools using data from the 2011–2012 Common Core Data (CCD) [39]. Specifically, NCES defines schools with 25.0% or fewer students eligible for FRPL as low poverty; schools with 25.1% to 50.0% as mid-low poverty; schools with 50.1% to 75.0% as mid-high poverty; and schools with more than 75.0% as high poverty.

Data on the racial and ethnic composition of students in each school came from the 2011–2012 CCD. It classified schools into one of four subgroups based on the racial and ethnic composition of students who attended the school: majority white; majority black; majority Hispanic; and diverse. Schools in which two-thirds of students identified as non-Hispanic, white were classified as majority white schools, whereas schools in which 50% or more of students identified as non-Hispanic, black were classified as majority black. Similarly, schools in which 50% or more students identified as Hispanic were classified as majority Hispanic. Schools that did not fit into any of these categories were classified as diverse [40]. Nine schools that were missing information on the racial and ethnic composition of students were omitted from analyses that explored differences by race/ethnicity subgroups.

### 2.5. Statistical Methods

Descriptive analyses were conducted to estimate the prevalence of disparities in the healthfulness of school food environments and the nutritional quality of NSLP lunches by poverty level and racial/ethnic composition. Differences across schools in each subgroup were flagged if statistically significant at an alpha of 0.05. All calculations were performed in R version 3.5.1 using the survey package [41,42], which accounted for the study’s clustered survey design. Pearson chi-square tests were used to test for statistical significance of differences in analyses related to school food environments. One-way analysis of variance (ANOVA) tests were used to test for statistical significance of differences in the nutritional quality of NSLP lunches across school types. For one-way ANOVA tests with a statistically significant result, post-hoc Tukey tests were performed to identify, which subgroups had statistically significantly different means from one another, adjusted for multiple hypothesis testing using the multcomp package [43,44]. A combination of visual inspection, assessments using skewness and kurtosis of residuals, and formal normality and homogeneity of variance tests (e.g., Shapiro–Wilks and Levene’s tests, respectively) were performed to ensure that the distributional assumptions of ANOVA were met. When characteristics deviated substantially from the normality assumptions of ANOVA, which was the case for five HEI component scores, Kruskal-Wallis tests were performed. Pairwise Wilcoxon rank-sum tests, adjusted for multiple hypothesis testing using a Benjamini–Hochberg correction, were performed for statistically significant Kruskal-Wallis tests [45].

## 3. Results

### 3.1. Sample Characteristics

Table 2 provides the weighted characteristics of the analytical samples for the analyses by poverty and race/ethnicity subgroups. Although 9 schools were omitted from the race/ethnicity subgroup analyses, there were no meaningful differences between the racial/ethnic analytical sample and the school poverty sample with regards to the distribution of schools by school level, school urbanicity, school size, or region. In both samples, approximately 60% of schools were classified as elementary schools, 18% as middle schools, and 22% as high schools. Approximately, 22% of schools were located in urban areas, while 43% were in suburban areas, and 35% were in rural areas. Nearly half (49%) of the schools in each sample were small schools, with fewer than 500 students enrolled, whereas only 12% of schools were large with more than 1000 or more students enrolled. Schools were roughly evenly distributed across seven geographic regions, with the highest proportion of schools located in the Midwest (19%).

### 3.2. Disparities in School Food Environments

#### 3.2.1. School Poverty Level

The proportion of schools differed significantly between poverty subgroups for three of the four characteristics related to the presence and sources of competitive foods. These included offering competitive foods, selling foods other than milk on an a la carte basis, and selling foods in vending machines (Table 3). In particular, 76% of high poverty schools offered competitive foods, compared to 85% of mid-high poverty schools, 87% of mid-low poverty schools, and 91% of low-poverty schools. About 63% of high poverty schools sold foods other than milk on an a la carte basis, which was about 10 percentage points lower than mid-high poverty schools and mid-low poverty schools (74% and 75%, respectively) and 20 percentage points lower than low poverty schools (83%). About 30% of mid-high and 23% of high poverty schools also reported selling foods in vending machines compared with 33% of mid-low poverty schools, and 36% of low poverty schools.

For characteristics related to school food policies and programs, the analysis revealed significant differences in the proportion of schools reporting a school wellness policy in addition to their SFAs’ wellness policy, participating in the FFVP, and belonging to an SFA with a pouring rights contract. One in four (26%) high poverty schools indicated having a school wellness policy in addition to their SFA wellness policy compared with 19% of mid-low poverty schools, 16% of low poverty schools, and 13% of mid-high poverty schools. Nearly 27% of high poverty schools reported participating in the FFVP, which was nearly double the proportion of low poverty and mid-low poverty schools (12% and 13%, respectively). Almost one-third (32%) of mid-high poverty schools were part of an SFA that had a pouring rights contract, compared with 28% of mid-low poverty schools, 22% of low poverty schools, and 17% of high poverty schools.

The only nutrition outreach activity that varied significantly by poverty level was related to sharing information about the school meal program with a nutrition advisory council. About 48% of high poverty schools reported sharing this information with a nutrition advisory council; the next highest percentage was among low poverty schools (43%), followed by mid-low poverty schools (35%), and mid-high poverty schools (33%).

#### 3.2.2. Racial/Ethnic Composition

The proportion of schools that offered competitive foods differed significantly based on the racial/ethnic composition of schools (Table 4). About 86% of majority white schools and 90% of diverse schools offered competitive foods, whereas, 77% of majority Hispanic and 68% of majority black schools did. About one-third of majority white and diverse schools (34% and 32%, respectively) sold foods in vending machines compared to one-fifth or fewer of majority black and majority Hispanic schools (21% and 14%, respectively). With respect to school food policies and programs, there were statistically significant differences in the distribution of schools whose SFA had a pouring rights contract and schools that reported having a school wellness policy in addition to an SFA wellness policy. About 30% of majority white schools were part of an SFA with a pouring rights contract; the next highest percentage was among majority black schools (23%), followed by diverse schools (22%), and majority Hispanic schools (11%). The percentage of majority black schools that reported having a school wellness policy in addition to an SFA wellness policy was roughly twice that of majority white schools and diverse schools (32% compared to 17% and 15%, respectively).

### 3.3. Disparities in the Nutritional Quality of NSLP Lunches

#### 3.3.1. School Poverty Level

There were no statistically significant differences by school poverty level in mean total HEI-2010 scores for NSLP lunches. Schools in each poverty subgroup achieved 81% to 82% of the maximum score (Table 5). Among the 12 HEI-2010 components, mean scores varied significantly across poverty subgroups only for total protein foods. Low poverty schools, on average, achieved 81% of the maximum score for this component, whereas the other subgroups averaged 90% to 91% of the maximum score.

#### 3.3.2. Racial/Ethnic Composition

There were no statistically significant differences across racial/ethnic subgroups in mean total HEI-2010 scores for NSLP lunches (Table 6). However, four significant results appeared among the component scores. Majority black schools received significantly higher scores for greens and beans (84%) in comparison to majority white schools and diverse schools (scores of 73% and 68%, respectively). NSLP lunches in majority white schools received a significantly higher score for total vegetables than diverse schools (85% of the maximum score versus 78%). NSLP lunches in majority Hispanic schools and diverse schools received significantly higher scores for sodium (indicating lower concentrations of sodium in the lunches) than lunches in majority white schools (scores of 35% and 33%, respectively, versus 24%).

## 4. Discussion

This analysis found evidence that school food environments varied by both the schools’ poverty level and racial/ethnic composition. Although findings for the two subgroups differed, the analysis consistently found statistically significant differences in the proportion of schools that offered competitive foods, those that had a school wellness policy in addition to an SFA wellness policy, and those that were part of an SFA with a pouring rights contract.

Prior to the passage of the HHFKA, competitive foods typically had low nutritional value [46,47] and several studies associated reduced access to competitive foods with healthier student diets [48,49,50,51,52], increased participation in the NSLP [53], and a reduced prevalence of obesity [52,54]. However, given that the Smart Snacks in School standards implemented as a result of the HHFKA sought to improve the nutritional quality of competitive foods available in schools, more research is needed to examine the relationship between access to competitive foods offered in schools under the new standards and the health of students who consume them.

This analysis showed that across schools in all subgroups, competitive foods were commonly available. However, higher proportions of low poverty, majority white, and diverse schools offered competitive foods than other types of schools. Higher proportions of schools in these subgroups also sold foods in vending machines than other schools. Together, these findings indicate that students in higher poverty schools and those with majority black and majority Hispanic students had less access to competitive foods than students in higher income, majority white, and diverse schools. This finding might suggest that high poverty, majority black, and majority Hispanic schools provide healthier school food environments than other school types; however, the SNMCS did not assess the nutritional quality of the competitive foods offered. Additionally, less than two-thirds of schools in all subgroups were part of an SFA that had fully implemented the Smart Snacks in School standards at the time of data collection. Thus, additional research is needed to examine the nutritional quality of competitive foods offered to students since the full implementation of the Smart Snacks in School standards to better understand whether disparities exist in the nutritional content of competitive foods offered in schools.

The results also indicate that higher proportions of high poverty, majority black, and majority Hispanic schools had both school wellness and SFA wellness policies. Research shows that having a school wellness policy in addition to a district policy improves school practices and reduces barriers in achieving wellness policy goals over time [55], providing further evidence that high poverty, majority black, and majority Hispanic schools had healthier school food environments than other school types.

With respect to differences in the nutritional quality of NSLP lunches (as measured by the HEI-2010), there were few differences across schools in each subgroup. On average, all schools, regardless of the composition of students, provided nutritious lunches, with total HEI-2010 scores ranging from 81% to 83% of the maximum score. This finding suggests that there are no disparities in the overall nutritional quality of NSLP lunches by school poverty level or race/ethnicity, which is encouraging given that low-income, black, and Hispanic children are at higher risk of obesity and other related health problems [56]. NSLP lunches in all types of schools were high in sodium, relative to the limit recommended in the DGA (scores ranging from 24% to 35% of the maximum score). However, results showed that the mean sodium scores for NSLP lunches in Hispanic and diverse schools were 10 percentage points higher than the score for majority white schools, indicating that Hispanic and diverse schools provided lower sodium meals, on average, than majority white schools. Research has shown that many children, particularly adolescents, consume sodium in excess of recommended amounts [57,58]. Excess sodium intake during childhood can have adverse health effects for children [59] as well as predisposing them to hypertension as adults [60] and increasing their risk for early development of cardiovascular disease [61]. Future research should explore the relationship between the nutritional quality of school meals and child health, controlling for participation and other student characteristics, to better understand the role that the school food environment plays in affecting students’ health outcomes.

Only a handful of studies have explored disparities in the healthfulness of school food environments in the United States [25,26,62,63,64]. Most of these studies relied on data collected prior to the passage of the HHFKA and used different metrics to measure the healthfulness of school food environments, making direct comparisons difficult. A study from Finkelstein et al. (2008) used data from a nationally representative sample of 400 public schools in 2005 to explore the relationship between racial/ethnic composition and household income of students, and school food environments and policies [63]. This study found only a few statistically significant differences in the healthfulness of school food environments by income and racial/ethnic composition of students; however, many of the relationships that did not achieve statistical significance were consistent with findings from this analysis. For example, Finkelstein et al. (2008) found the mean school food environment and policy score was higher (indicating healthier) among schools with a higher percentage of non-white students, although the *p*-value (*p* = 0.06) was slightly above the threshold of 0.05 used in the study to identify statistically significant results. That study also found that 50% of low poverty schools sold foods in vending machines, compared with 47% of middle poverty schools, and 39% of high poverty schools, which is consistent with the trends observed in this analysis. Although Finkelstein et al. (2008) did not directly explore the proportion of schools with a school wellness policy in addition to an SFA wellness policy, they also found a greater proportion of high poverty schools (50%) reported having a wellness policy than middle and lower poverty schools (37% and 40%, respectively).

Balaji et al. (2010) examined variation in school health policies and programs using data from the 2006 School Health Policies and Programs Study [62]. They found schools with higher percentages of FRPL-eligible students were significantly less likely to offer branded fast food to students. This is consistent with trends observed in the current analysis, although none of the differences across poverty subgroups were statistically significant. Balaji et al. (2010) found no significant differences in the proportion of schools that participated in nutrition promotion activities by school poverty level or racial/ethnic composition; this is also consistent with findings from this analysis.

Two studies have assessed changes in the foods offered in NSLP lunches and the prevalence of disparities before and after the passage of the HHFKA [25,26]. One study by Terry-McElrath et al. surveyed about 600 middle and high schools on the frequency with which they offered sugar sweetened beverages, candy or regular-fat snacks, higher fat and nonfat milks, French fries, whole grains, and fruits and vegetables in NSLP lunches [19]. This study found significant improvements in the healthfulness of NSLP lunches offered to middle and high school students after the passage of the HHFKA, and reductions in racial/ethnic disparities. They found few disparities in the nutritional content of NSLP lunches based on school poverty level (as measured by the percentage of students qualifying for free lunch) before the passage of the HHFKA and determined that disparities continued to decrease after the HHFKA was in place. A second study by Turner et al. explored changes in the frequency with which a nationally representative sample of elementary schools offered foods such as different types of milks, fruits, vegetables, whole grains, and other items [20]. Their findings showed that although the healthfulness of NSLP lunch offerings increased significantly over time, disparities based on race/ethnicity and FRPL status persisted for some types of foods. Turner et al. found majority black or Latino schools were significantly less likely to offer fresh fruit than predominantly white schools, and salad was less available in schools with higher rates of FRPL participation than in schools with lower rates. The research from Terry-McElrath et al. and Turner et al. show that since the passage of the HHFKA, NSLP lunches generally offer healthy foods; however, there are some disparities in the types of food offered across schools with different poverty levels and racial/ethnic compositions. Findings from the current analysis suggest the overall nutritional quality of NSLP lunches did not differ by poverty level or racial/ethnic composition. Reasons for the different findings could stem from differences in the unit of analysis and scope of the studies. Terry-McElrath et al. and Turner et al. examined the types of foods offered in NSLP lunches, but this analysis focused on the overall nutritional quality of the meals using the HEI-2010. In addition, the current analysis reflects all school levels, whereas the other studies were restricted to specific grade levels.

It is important to note key limitations of the analyses presented here. First, because the subgroup analyses did not control for additional school or SFA characteristics, we cannot be certain the differences that emerged were due to the socioeconomic or racial/ethnic composition of students rather than other reasons, such as differences in school size, urbanicity, or school level. Second, although it is common practice to use the percentage of FRPL-eligible students as a proxy for school poverty status, it is possible this variable did not adequately capture differences in income or wealth across schools. As a result, had this analysis used an alternative measure for poverty, different results might have emerged. Third, because the SNMCS did not collect information needed to assess the nutritional quality of competitive foods sold by schools, it was not possible to assess compliance with the Smart Snacks in School standards or disparities in the nutritional quality of competitive foods. Finally, the SNMCS data were collected only two years after the implementation of the updated nutrition standards for school meals, which required schools to make major changes to their school meal programs. The healthfulness of school food environments and nutritional quality of NSLP lunches could continue to change over time as schools continue to adapt their menu planning, food purchasing, and food preparation methods to meet the updated standards. Therefore, additional research should assess whether disparities have changed since school year 2014–2015.

## 5. Conclusions

Past research has found limited evidence of disparities in the healthfulness of school food environments in the United States, but few studies have explored the prevalence of these disparities since the passage of the HHFKA. This analysis provides evidence that after the passage of the HHFKA, several aspects of the school food environment vary based on the socioeconomic and racial/ethnic composition of students in schools. The results further suggest high poverty and majority black and majority Hispanic schools tend to have more healthful school food environments than other school types. At the same time, the analysis did not find strong evidence to suggest the nutritional quality of NSLP lunches differed greatly by school type. Because the healthfulness of the school food environment and the nutritional quality of meals is likely to change over time as regulations are modified and schools implement changes, it is important to examine disparities on an ongoing basis.

## Figures and Tables

**Table 1:** Healthy Eating Index-2010 components and standards for scoring.

	Maximum Score	Standard for Maximum Score ^a^	Standard for Minimum Score of Zero ^a^
**Adequacy Components (Higher Scores Reflect Higher Concentrations in NSLP ^l^ Lunches)**			
Total fruit ^b^	5	≥0.8 c equivalent per 1000 kcal	No fruit
Whole fruit ^c^	5	≥0.4 c equivalent per 1000 kcal	No whole fruit
Total vegetables ^d^	5	≥1.1 c equivalent per 1000 kcal	No vegetables
Greens and beans ^d^	5	≥0.2 c equivalent per 1000 kcal	No dark green vegetables, beans, or peas
Whole grains	10	≥1.5 oz equivalent per 1000 kcal	No whole grains
Dairy ^e^	10	≥1.3 c equivalent per 1000 kcal	No dairy
Total protein foods ^f^	5	≥2.5 oz equivalent per 1000 kcal	No protein foods
Seafood and plant proteins ^f,g^	5	≥0.8 oz equivalent per 1000 kcal	No seafood or plant proteins
Fatty acids ^h^	10	(PUFAs ^i^ + MUFAs ^j^)/saturated fatty acids ≥ 2.5	(PUFAs + MUFAs)/saturated fatty acids ≤ 1.2
**Moderation components (higher scores reflect lower concentrations in NSLP lunches)**			
Refined grains	10	≤1.8 oz equivalent per 1000 kcal	≥4.3 oz equivalent per 1000 kcal
Sodium	10	≤1.1 g per 1000 kcal	≥2.0 g per 1000 kcal
Empty calories ^k^	20	≤19% of energy	≥50% of energy
Total Score	100		

Source: Adapted from USDA 2013 [34]. ^a^ Concentrations between the minimum and maximum standard are scored proportionately. Higher scores reflect higher nutritional quality. ^b^ Includes 100 percent fruit juice. ^c^ Includes all forms except juice. ^d^ Includes any beans and peas not counted as Total Protein Foods. ^e^ Includes all milk products, such as fluid milk, yogurt, cheese, and fortified soy beverages. ^f^ Beans and peas are included here (and not with vegetables) when the Total Protein Foods standard is otherwise not met. ^g^ Includes seafood, nuts, seeds, soy products (other than beverages) as well as beans and peas counted toward Total Protein Foods. ^h^ Ratio of poly- and monounsaturated fatty acids (PUFAs and MUFAs) to saturated fatty acids. ^i^ PUFA, polyunsaturated fatty acid. ^j^ MUFA, monounsaturated fatty acid. ^k^ Kcals from solid fats and added sugars. ^l^ NSLP, National School Lunch Program. School lunches do not include alcohol.

**Table 2:** Sample characteristics.

	Analysis: School Poverty (*n* = 1207)		Analysis: Race/Ethnicity (*n* = 1198)	
	%	95% CI	%	95% CI
**School Level**				
Elementary School	59.7	(57.4, 61.8)	59.9	(57.7, 62.1)
Middle School	18.2	(16.8, 19.8)	17.8	(20.4, 24.2)
High School	22.1	(20.3, 24.1)	22.2	(16.4, 19.4)
**School Urbanicity**				
Urban	21.8	(18.1, 26.0)	21.9	(18.2, 26.1)
Suburban	43.3	(38.6, 48.2)	43.1	(38.4, 48.0)
Rural	34.9	(30.5, 39.6)	35.0	(30.5, 39.7)
**School Size**				
Small (fewer than 500 students)	48.7	(44.5, 53.0)	48.9	(44.6, 53.2)
Medium (500 to 999 students)	39.3	(35.2, 43.5)	39.3	(35.3, 43.6)
Large (1000 or more students)	12.0	(10.3, 13.9)	11.8	(10.1, 13.7)
**Region**				
Midwest	18.8	(15.1, 23.2)	18.9	(15.2, 23.3)
Southeast	16.4	(12.8, 20.8)	16.5	(12.9, 20.9)
Western	16.6	(13.0, 21.1)	16.6	(12.9, 21.0)
Southwest	14.4	(11.1, 18.5)	14.4	(11.0, 18.5)
Mountain Plains	13.2	(9.5, 18.0)	13.2	(9.5, 18.0)
Mid-Atlantic	13.3	(10.2, 17.1)	13.1	(10.0, 16.9)
Northeast	7.4	(5.2, 10.4)	7.4	(5.2, 10.4)

CI, confidence interval

**Table 3:** Characteristics of school food environments, by school poverty.

		Low Poverty (*n* = 226)		Mid-Low Poverty (*n* = 376)	Mid-High Poverty (*n* = 330)		High Poverty (*n* = 275)		*p*-Value
	%	95% CI	%	95% CI	%	95% CI	%	95% CI	
**Presence and Sources of Competitive Foods**									
Offers competitive foods	91.4	(86.3, 96.6)	86.5	(81.9, 91.2)	85.2	(79.8, 90.6)	75.5	(68.2, 82.9)	<0.01 *
Sells foods other than milk on an a la carte basis	83.1	(75.8, 90.3)	74.7	(68.6, 80.9)	73.5	(66.6, 80.4)	62.6	(54.6, 70.5)	<0.01 *
Sells foods in a vending machine	36.3	(29.7, 42.9)	32.8	(26.8, 38.8)	30.0	(23.7, 36.4)	22.9	(16.8, 29.0)	0.03 *
Sells foods in a school store, snack bar, food cart, kiosk, or fund-raiser	15.8	(9.3, 22.2)	17.8	(12.0, 23.6)	11.6	(7.5, 15.7)	17.4	(10.2, 24.6)	0.36
**School Food Policies and Programs**									
Operates a school garden	12.4	(5.7, 19.1)	3.6	(1.4, 5.9)	8.0	(3.5, 12.4)	8.0	(3.7, 12.2)	0.06
Participates in FFVP ^a^	12.2	(6.0, 18.5)	13.3	(8.0, 18.6)	19.9	(12.9, 27.0)	26.6	(19.6, 33.7)	0.01 *
Participates in Farm to School	22.3	(13.9, 30.8)	17.0	(10.7, 23.2)	15.9	(8.5, 23.4)	15.2	(8.6, 21.8)	0.58
SFA offers branded foods	13.4	(6.6, 20.2)	10.0	(5.8, 14.1)	9.3	(4.7, 13.9)	4.5	(0.7, 8.2)	0.08
SFA has pouring rights contract	22.2	(12.9, 31.5)	28.0	(20.5, 35.6)	31.9	(23.0, 40.9)	17.0	(10.7, 23.4)	0.02 *
Has a school wellness policy in addition to SFA wellness policy	15.9	(9.7, 22.0)	19.0	(13.1, 24.8)	12.9	(7.9, 17.9)	25.5	(18.0, 32.9)	0.02 *
SFA fully implemented Smart Snacks in School standards	61.7	(50.0, 72.2)	63.2	(54.4, 71.1)	62.4	(52.7, 71.1)	53.3	(44.1, 62.3)	0.34
SFA has standards that exceed Smart Snacks in School standards	22.9	(15.6, 32.4)	29.4	(22.8, 37.0)	36.9	(28.5, 46.3)	28.5	(21.3, 37.1)	0.13
**Nutrition Promotion and Outreach**									
Shared information about school meal program with nutrition advisory council	43.1	(32.5, 53.8)	34.7	(27.0, 42.4)	32.8	(25.2, 40.3)	47.5	(39.2, 55.8)	0.03 *
Conducted a nutrition education activity in the classroom or food service area	50.2	(39.7, 60.7)	44.2	(36.9, 51.5)	54.9	(46.2, 63.6)	54.6	(46.4, 62.9)	0.20
Encouraged students to select fruit	88.7	(83.2, 94.3)	90.7	(86.9, 94.5)	87.1	(81.7, 92.5)	90.5	(85.3, 95.7)	0.67
Involved students in planning of menus	45.0	(34.6, 55.5)	45.5	(37.7, 53.3)	40.1	(32.5, 47.7)	37.2	(29.1, 45.3)	0.41
Sought student input into vegetable offerings	46.0	(35.6, 56.4)	48.7	(40.9, 56.5)	50.0	(41.9, 58.1)	50.0	(41.7, 58.3)	0.93

* *p* < 0.05. ^a^ Only elementary schools can participate in the FFVP. Middle and high schools are included in the analysis and are treated as if they do not offer the FFVP. CI, confidence interval; SFA, school food authority; FFVP—Fresh Fruit and Vegetable Program.

**Table 4:** Characteristics of school food environments, by racial/ethnic composition.

	Majority White (*n* = 643)		Majority Black (*n* = 89)		Majority Hispanic (*n* = 156)		Diverse (*n* = 310)		*p*-Value
	%	95% CI	%	95% CI	%	95% CI	%	95% CI	
**Presence and Sources of Competitive Foods**									
Offers competitive foods	85.5	(81.6, 89.5)	67.6	(52.2, 83.0)	77.3	(67.4, 87.1)	90.0	(85.5, 94.6)	<0.01 *
Sells foods other than milk on an a la carte basis	75.3	(70.5, 80.1)	67.1	(52.3, 81.9)	60.2	(48.8, 71.6)	74.7	(67.1, 82.3)	0.07
Sells foods in a vending machine	33.9	(29.7, 38.0)	21.4	(9.9, 32.9)	14.2	(9.0, 19.4)	32.2	(25.4, 39.1)	<0.01 *
Sells foods in a school store, snack bar, food cart, kiosk, or fund-raiser	14.3	(10.4, 18.3)	19.8	(3.3, 36.2)	18.8	(9.5, 28.0)	15.6	(10.0, 21.3)	0.73
**School Food Policies and Programs**									
Operates a school garden	7.6	(4.7, 10.5)	5.1	(0.0, 11.0)	10.3	(3.1, 17.6)	6.6	(2.9, 10.4)	0.67
Participates in FFVP ^a^	19.6	(15.2, 23.9)	26.7	(13.2, 40.1)	21.7	(10.9, 32.4)	12.6	(6.8, 18.4)	0.17
Participates in Farm to School	17.2	(11.8, 22.5)	21.3	(9.3, 33.3)	14.2	(4.8, 23.6)	16.1	(9.6, 22.6)	0.81
SFA offers branded foods	7.7	(4.4, 10.9)	3.4	(0.0, 7.7)	9.9	(2.4, 17.5)	12.5	(6.6, 18.5)	0.18
SFA has pouring rights contract	30.2	(23.4, 37.1)	23.4	(11.1, 35.7)	11.4	(2.7, 20.1)	22.3	(14.5, 30.2)	0.02 *
Has a school wellness policy in addition to SFA wellness policy	16.6	(12.7, 20.4)	31.7	(17.5, 45.9)	25.4	(14.9, 35.9)	14.8	(9.2, 20.4)	0.02 *
SFA fully implemented Smart Snacks in School standards	59.6	(52.0, 66.8)	58.3	(40.4, 74.3)	53.2	(40.5, 65.5)	64.8	(55.2, 73.3)	0.57
SFA has standards that exceed Smart Snacks in School standards	29.8	(23.6, 36.8)	29.3	(16.3, 46.9)	32.9	(22.4, 45.5)	29.4	(21.4, 38.8)	0.97
**Nutrition Promotion and Outreach**									
Shared information about school meal program with nutrition advisory council	35.2	(29.5, 40.9)	52.2	(38.7, 65.6)	43.9	(31.7, 56.1)	39.5	(30.7, 48.2)	0.12
Conducted a nutrition education activity in the classroom or food service area	49.1	(42.6, 55.5)	54.9	(40.8, 68.9)	50.4	(38.6, 62.3)	54.2	(45.5, 62.8)	0.75
Encouraged students to select fruit	90.3	(87.1, 93.5)	87.2	(76.6, 97.9)	96.3	(93.4, 99.3)	84.4	(78.0, 90.8)	0.05
Involved students in planning of menus	43.0	(36.7, 49.3)	42.0	(28.1, 56.0)	37.5	(25.8, 49.2)	41.2	(32.8, 49.6)	0.87
Sought student input into vegetable offerings	50.1	(44.1, 56.2)	49.1	(34.7, 63.5)	48.1	(36.3, 59.9)	47.0	(38.7, 55.3)	0.94

* *p* < 0.05. ^a^ Only elementary schools can participate in the FFVP. Middle and high schools are included in the analysis and are treated as if they do not offer the FFVP. CI, confidence interval; SFA, school food authority; FFVP, Fresh Fruit and Vegetable Program.

**Table 5:** Mean HEI-2010 scores for NSLP lunches, expressed as a percentage of the maximum possible scores, by school poverty level.

	Low Poverty (*n* = 226)		Mid-Low Poverty (*n* = 376)		Mid-High Poverty (*n* = 330)		High Poverty (*n* = 275)		*p*-Value *
**Adequacy Components (Higher Scores Reflect Higher Concentrations in NSLP Lunches)**									
^∞^ Total fruit	100	(100, 100)	100	(100, 100)	100	(99.0, 100)	100	(98.9, 100)	0.37
^∞^ Whole fruit	100	(100, 100)	100	(100, 100)	100	(100, 100)	100	(100, 100)	0.98
Total vegetables	79.2	1.8	81.7	1.5	84.1	1.5	83.8	1.9	0.17
Greens and beans	65.8	2.9	72.8	2.0	74.0	2.5	74.6	2.7	0.11
^∞^ Whole grains	100	(100, 100)	100	(100, 100)	100	(100, 100)	100	(100, 100)	0.19
Dairy	100	0.0	100	0.0	100	0.0	100	0.0	n.a.
Total protein foods	81.4	1.8	90.9 ^a^	0.8	91.0 ^a^	0.9	90.2 ^a^	1.1	<0.01
Seafood and plant proteins	45.9	2.6	49.6	2.1	50.3	2.4	49.5	3.0	0.61
Fatty acids	61.3	2.7	66.9	1.5	62.8	1.8	61.3	2.1	0.06
**Moderation Components (Higher Scores Reflect Lower Concentrations in NSLP Lunches)**									
^∞^ Refined grains	100	(100, 100)	100	(100, 100)	100	(100, 100)	100	(100, 100)	0.30
Sodium	31.4	2.2	26.8	1.8	26.7	1.7	29.4	1.9	0.31
^∞^ Empty calories	100	(93.2, 100)	100	(92.5, 100)	100	(92.6, 100)	100	(93.3, 100)	0.98
**Total Score**	80.8	0.5	81.9	0.5	82.1	0.4	82.0	0.5	0.22

**Note:** HEI scores are expressed as a percentage of maximum possible scores. Estimates are means and standard errors, or medians and (interquartile ranges) where specified by ^∞^; * One-way ANOVA and Kruskal-Wallis for non-parametric variables; superscript letters indicate a statistically significant difference (adjusted *p* < 0.05). ^a^ Comparison between this group and low poverty. n.a. = given the lack of variation in the mean scores for dairy across subgroups, statistical testing was not performed. HEI-2010, Healthy Eating Index-2010; NSLP, National School Lunch Program; SE, standard error.

**Table 6:** Mean HEI-2010 scores for NSLP lunches, expressed as a percentage of the maximum possible scores, by racial/ethnic composition.

	Majority White (*n* = 643)		Majority Black (*n* = 89)		Majority Hispanic (*n* = 156)		Diverse (*n* = 310)		*p*-Value *
**Adequacy Components (Higher Scores Reflect Higher Concentrations in NSLP Lunches)**									
^∞^ Total fruit	100	(97.8, 100)	100	(100, 100)	100	(98.4, 100)	100	(100, 100)	0.08
^∞^ Whole fruit	100	(100, 100)	100 ^a^	(100, 100)	100	(100, 100)	100 ^b^	(100, 100)	<0.01
Total vegetables	85.4	1.0	83.1	3.5	80.3	2.8	77.7 ^a^	1.8	<0.01
Greens and beans	73.1	1.7	84.2 ^a^	3.8	72.1	3.9	67.7 ^b^	2.6	<0.01
^∞^ Whole grains	100.0	(100, 100)	100	(100, 100)	100	(100, 100)	100	(100, 100)	0.99
Dairy	100	0.0	100	0.0	100	0.0	100	0.0	n.a.
Total protein foods	88.6	0.8	90.1	2.1	90.5	1.5	89.5	1.2	0.70
Seafood and plant proteins	48.7	1.7	40.5	5.2	55.6	4.0	49.9	2.7	0.14
Fatty acids	63.4	1.4	61.9	3.1	60.2	3.5	65.2	1.9	0.58
**Moderation Components (Higher Scores Reflect Lower Concentrations in NSLP Lunches)**									
^∞^ Refined grains	100	(100, 100)	100	(98.3, 100)	100	(100, 100)	100	(100, 100)	0.27
Sodium	24.0	1.2	29.4	3.3	34.6 ^a^	2.8	33.4 ^a^	1.9	<0.01
^∞^ Empty calories	98.9	(92.1, 100)	100.0	(95.6, 100)	100.0	(94.8, 100)	100.0	(94.6, 100)	0.07
**Total Score**	81.4	0.4	82.4	0.6	82.7	0.8	82.1	0.5	0.22

**Note:** HEI scores are expressed as a percentage of maximum possible scores. Estimates are means and standard errors, or medians and (interquartile ranges) where specified by ^∞^; * One-way ANOVA and Kruskal-Wallis for non-parametric variables; superscript letters indicate a statistically significant difference (adjusted *p* < 0.05). ^a^ Comparison between this group and majority white. ^b^ Comparison between this group and majority black. n.a. = given the lack of variation in the mean scores for dairy across subgroups, statistical testing was not performed. HEI-2010, Healthy Eating Index-2010, NSLP, National School Lunch Program; SE, standard error.
